# Did Genetic Drift Drive Increases in Genome Complexity?

**DOI:** 10.1371/journal.pgen.1001080

**Published:** 2010-08-26

**Authors:** Kenneth D. Whitney, Theodore Garland

**Affiliations:** 1 Department of Ecology and Evolutionary Biology, Rice University, Houston, Texas, United States of America; 2 Department of Biology, University of California Riverside, Riverside, California, United States of America; Yale University, United States of America

## Abstract

Mechanisms underlying the dramatic patterns of genome size variation across the tree of life remain mysterious. Effective population size (*N_e_*) has been proposed as a major driver of genome size: selection is expected to efficiently weed out deleterious mutations increasing genome size in lineages with large (but not small) *N_e_*. Strong support for this model was claimed from a comparative analysis of *N_e_u* and genome size for ≈30 phylogenetically diverse species ranging from bacteria to vertebrates, but analyses at that scale have so far failed to account for phylogenetic nonindependence of species. In our reanalysis, accounting for phylogenetic history substantially altered the perceived strength of the relationship between *N_e_u* and genomic attributes: there were no statistically significant associations between *N_e_u* and gene number, intron size, intron number, the half-life of gene duplicates, transposon number, transposons as a fraction of the genome, or overall genome size. We conclude that current datasets do not support the hypothesis of a mechanistic connection between *N_e_* and these genomic attributes, and we suggest that further progress requires larger datasets, phylogenetic comparative methods, more robust estimators of genetic drift, and a multivariate approach that accounts for correlations between putative explanatory variables.

## Author Summary

Genome size (the amount of nuclear DNA) varies tremendously across organisms but is not necessarily correlated with organismal complexity. For example, genome sizes just within the grasses vary nearly 20-fold, but large-genomed grass species are not obviously more complex in terms of morphology or physiology than are the small-genomed species. Recent explanations for genome size variation have instead been dominated by the idea that population size determines genome size: mutations that increase genome size are expected to drift to fixation in species with small populations, but such mutations would be eliminated in species with large populations where natural selection operates at higher efficiency. However, inferences from previous analyses are limited because they fail to recognize that species share evolutionary histories and thus are not necessarily statistically independent. Our analysis takes a phylogenetic perspective and, contrary to previous studies, finds no evidence that genome size or any of its components (e.g., transposon number, intron number) are related to population size. We suggest that genome size evolution is unlikely to be neatly explained by a single factor such as population size.

## Introduction

The vast array of genome sizes is a pattern that begs for explanation [1], [2]. Haploid (1C) genome size (measured either in base pairs or mass, where 10^6^ Kb ≈1 picogram) spans eight orders of magnitude: the known eukaryotic range is ≈2,249–978,000,000 Kb [3], while Archaea and Bacteria range from 491–5,751 Kb and 76–13,034 Kb, respectively [4].

Lynch and colleagues [5]–[7] have argued strongly for a central role for nonadaptive processes such as mutation and drift in the evolution of genome size and complexity. In contrast to proposed neutral and adaptive models of genome size evolution (see, e.g. [8], [9]), they outline a model positing that mutations increasing genome size are slightly deleterious. Under this model, lineages differ in effective population size (*N_e_*) and, as a result, differ in the efficacy with which natural selection will counteract genome expansion. Thus, lineages with small *N_e_* will experience drift towards larger genomes [7]. As support for their argument, they presented a comparative analysis of roughly 30 taxa, ranging from bacteria to angiosperms, fungi, and mammals. Among these taxa, they reported a statistically significant negative relationship between *N_e_u* (a composite parameter including effective population size and nucleotide mutation rate) and genome size. Strikingly, the relationship was quite strong: 66% of the variation in genome size was explained by *N_e_u* [7]. This is truly an astounding result, considering the widely divergent selective regimes, life histories, and modes of reproduction found across these diverse organisms.

The Lynch & Conery model has sparked intense interest and >330 citations. Some objections on theoretical and methodological grounds have been voiced. Charlesworth and Barton [10] point out that *N_e_* is confounded with many different aspects of organismal biology (e.g., developmental rate, body size), and thus that both *N_e_* and genome size may be correlated effects of one or more other causal factors. Daubin and Moran [11] outline several objections, including that taxon differences in mutation rates make *N_e_u* a poor proxy for *N_e_* that estimates of *N_e_* from silent-site nucleotide diversity in bacteria (as in [7]) are skewed by population subdivision and cryptic species, and further that such *N_e_* estimates are overly sensitive to recent evolutionary history. Nevertheless, the idea that *N_e_* drives genome size and complexity seems to have gained acceptance [12]–[14], with some going so far as to characterize it as “the principal explanatory framework for understanding the evolution of genome organization” ([12], p. 303).

**Figure 1:**
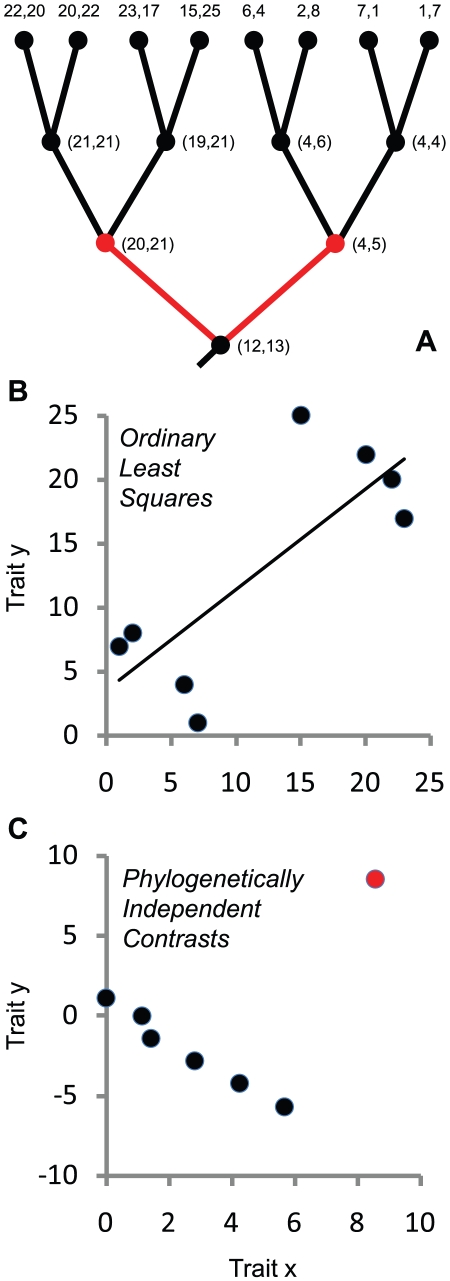
Ignoring phylogenetic history can lead to incorrect conclusions about the nature of evolutionary associations between traits. In this hypothetical example, eight species have been measured for two traits, x and y, as indicated by pairs of values at the tips of the phylogenetic tree (A). Ordinary least-squares linear regression (OLS) indicates a statistically significant positive relationship (B; *r^2^* = 0.62, *P* = 0.02), potentially leading to an inference of a positive evolutionary association between x and y. However, inspection of the scatterplot (B) in relation to the phylogenetic relationships of the species (A) indicates that the association between x and y is *negative* for the four species within each of the two major lineages. Regression through the origin with phylogenetically independent contrasts (computed using [34] and setting all branches to length 1.0), which is equivalent to phylogenetic generalized least squares (PGLS) analysis, accounts for the nonindependence of species and indicates no overall evolutionary relationship between the traits (C, standardized contrasts, *r^2^* = 0.01, *P* = 0.82; basal contrast indicated in red). The apparent pattern across species was driven by positively correlated trait change only at the basal split of the phylogeny; throughout the rest of the phylogeny, the traits mostly changed in *opposite* directions (A; basal contrast in red). Notes: In A, the estimated nodal values for both traits are shown in parentheses. These are intermediate steps in the independent contrasts algorithm and are not to be taken as optimal estimates of the states at internal nodes; rather, they are a type of “local parsimony” estimate (except the estimate at the basal node, which is equivalent to the estimate under squared-change parsimony). Contrasts are taken between sister nodes on a phylogeny, not along each branch segment [15], [16], [18].

Here, we argue that such conclusions are premature without phylogenetic comparative analyses of genome size evolution. When species are used as data points, relationships between raw values of any two traits (e.g., *N_e_* and genome size) are difficult to interpret, as shared phylogenetic history means that assumptions of statistical independence are likely to be violated [15]–[17]. Special methods are required to recover independence of observations and to test for evolutionary associations between traits. Frequently, conventional (nonphylogenetic) analyses overestimate the strength of the association between traits relative to phylogenetic methods [18]. In an extreme case, a strong correlation in the raw data can be driven by a single association at the base of the phylogenetic tree, e.g., it can reflect a single instance of correlated change in the traits, followed by uncorrelated changes and/or stasis in trait values during subsequent evolutionary history (Figure 1). In this study, we revisit the Lynch & Conery dataset with a phylogenetic perspective, taking advantage of new phylogenetic data and analysis tools.

## Results

### Model fitting

**Figure 2:**
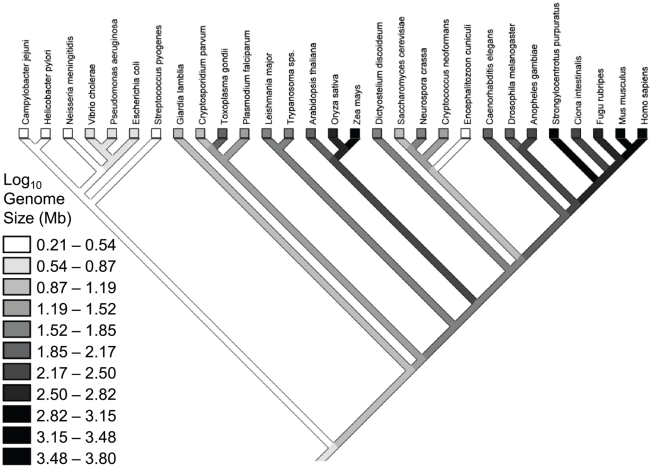
Phylogeny for the species in the Lynch & Conery dataset [**7**], with a reconstruction of genome sizes. (See Materials and Methods).

A phylogenetic topology and reconstruction of genome sizes is presented in Figure 2, illustrating that close relatives have similar genome sizes. Initial simple linear regressions of genome size on *N_e_u* explored four branch length models and found that the phylogenetic generalized least squares (PGLS) model with all branches  = 1.0 provided a better fit than the nonphylogenetic ordinary least squares (OLS) model (Table 1). Subsequent analyses therefore used branch lengths of 1.0. For all variables except intron number, phylogenetic models (PGLS) exhibited better fit than nonphylogenetic (OLS) models (Table 1). For genome size and gene number, estimation of the Ornstein-Uhlenbeck transformation parameter *d* indicated substantial phylogenetic signal (*d* = 1.31 and 1.16, respectively), and the resulting RegOU models fit significantly better than the OLS models (ln likelihood ratio tests (LRTs), χ^2^ = 5.88, *P* = 0.015 and χ^2^ = 7.90, *P* = 0.005, respectively). In comparing the two phylogenetic models, the RegOU model did not produce significantly better fit vs. PGLS (LRTs, χ^2^ = 1.84, *P* = 0.175 and χ^2^ = 0.46, *P* = 0.498 for genome size and gene number, respectively).

**Table 1:** Relationships between *N_e_u* and genomic attributes in nonphylogenetic (OLS) and phylogenetic (PGLS, RegOU) models.

Model	Dependent variable	ln Max Likelihood	*N*	*b*	*r* ^2^	*P* for regression
***Ordinary Least Squares (OLS)***						
	Genome Size (Mb)	−25.53	29	−1.17	0.64†	**<0.001**
	Gene Number	−07.81	28	−0.54	0.56	**<0.001**
	Half-life of Gene Duplicates	25.87	9	−0.03	0.52	**0.028**
	Intron Size	−09.60	15	−0.68	0.40	**0.011**
	Intron Number	−23.40	15	−1.06	0.21	0.084
	Transposons (number)	−35.49	18	−2.27	0.35	**0.010**
	Transposons (fraction of genome)	−12.06	18	−0.56	0.31	**0.017**
***Phylogenetic Generalized Least Squares (PGLS)***						
	Genome Size (Mb)	−23.51	29	−0.33	0.08	0.137
	Gene Number	−04.09	28	−0.15	0.07	0.187
	Half-life of Gene Duplicates	23.62	9	−0.01	0.13	0.335
	Intron Size	−09.33	15	−0.36	0.13	0.187
	Intron Number	−23.84	15	−0.75	0.09	0.291
	Transposons (number)	−33.83	18	−0.29	0.01	0.707
	Transposons (fraction of genome)	−11.52	18	−0.07	0.01	0.740
***Phylogenetic Regression under an Ornstein-Uhlenbeck Process (RegOU)***						
	Genome Size (Mb)	−22.59*	29	−0.20	0.04	0.328
	Gene Number	−03.86*	28	−0.12	0.04	0.282

Log_10_-transformed dependent variables were regressed on log_10_(*N_e_u*). Phylogenetic models used arbitrary branch lengths of 1.0 (see Materials and Methods). Note that *r^2^* values are not comparable across OLS, PGLS, and RegOU models. Asterisks indicate RegOU models with significantly better fit than OLS models, based on ln likelihood ratio tests (see Results); *b* = regression slope; significant *P*-values are in bold.

**†:** Lynch & Conery [7] reported *r^2^* = 0.659; the discrepancy apparently arises because their analysis used 30 species, only 29 of which were reported in their online supplement.

### Phylogenetic regressions do not detect relationships between *N_e_u* and genomic attributes

**Figure 3:**
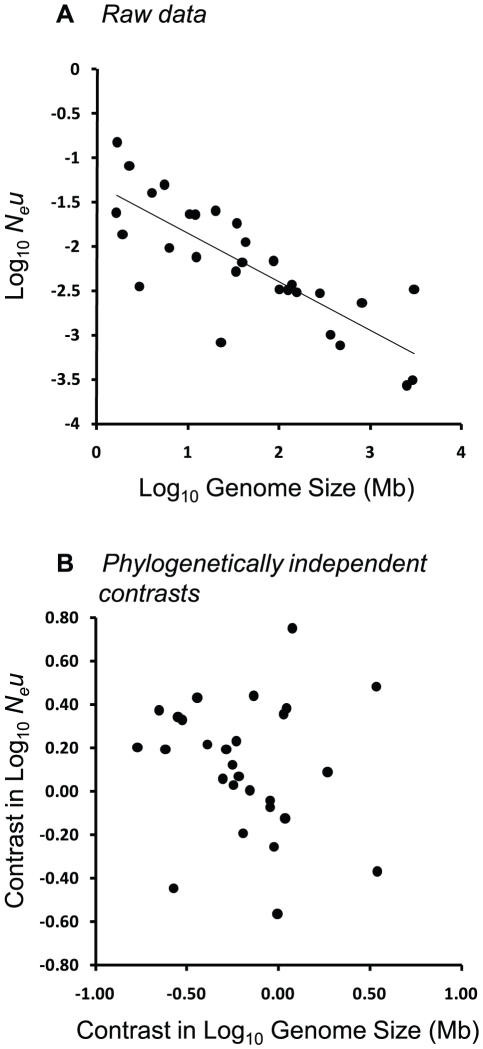
Relationship between *N_e_u* and genome size across 22 eukaryotic and 7 prokaryotic species from the dataset of Lynch & Conery [**7**]. (A) Ordinary least squares regression (OLS); *r^2^* = 0.64, *P*<0.0001. (B) Standardized phylogenetically independent contrasts (equivalent to PGLS) using branch lengths of 1.0; *r^2^* = 0.08, *P* = 0.138. Values have been “positivized” on the x-axis [35].

Although there were strong negative relationships between *N_e_u* and six of the seven genomic attributes in nonphylogenetic regressions, the patterns disappeared when phylogenetic models were applied (Table 1). For example, the strong negative relationship between *N_e_u* and genome size (OLS, *P*<0.001, Figure 3A) was replaced with a nonsignificant relationship under better-fitting phylogenetic models (PGLS, *P* = 0.137, Figure 3B; RegOU, *P* = 0.328). Similar patterns were evident for gene number, the half-life of gene duplicates, intron size, intron number, transposon number, and transposon fraction (Table 1).

## Discussion

Accounting for phylogenetic history substantially altered the perceived strength of the relationship between *N_e_u* and genomic attributes. In phylogenetic analyses, there were no consistent evolutionary associations between *N_e_u* and gene number, intron size, intron number, the half-life of gene duplicates, transposon number, transposons as a fraction of the genome, or overall genome size. Thus, a phylogenetically controlled reanalysis of the Lynch & Conery dataset [7] does not support the conclusion that *N_e_* drives genome size patterns across the tree of life.

The few existing comparative analyses of more phylogenetically restricted datasets either do not support or provide only equivocal support for the Lynch & Conery model. Whitney et al. [19] conducted a phylogenetically controlled analysis of 205 species of seed plants and found no association between *N_e_* and genome size. Kuo et al. [20] analyzed 42 paired bacterial genomes, using the efficacy of purifying selection in coding regions to quantify genetic drift. Bacterial taxa experiencing greater levels of genetic drift – implying a smaller evolutionary *N_e_* – had smaller genomes, a pattern opposite that predicted by the Lynch & Conery model as articulated in [7]. Finally, in putative support of the model, Yi & Streelman [21] reported a significant negative relationship between *N_e_* and genome size in a phylogenetically corrected analysis of 33 species of ray-finned fish. However, this analysis has been challenged as artifactual. Gregory & Witt [22] argue that Pleistocene population bottlenecks and polyploidy shaped both *N_e_* and genome size of fishes in such a way as to generate a non-causal correlation between *N_e_* and genome size in this particular dataset.

Future investigations of the role of genetic drift in determining genome size across the tree of life would benefit from several approaches. First, utilizing phylogenetic comparative methods, for which we advocate here, is an important step towards drawing robust inferences from species-level comparative analyses. Second, larger datasets would certainly increase confidence in our interpretations. While statistically nonsignificant, we note the relationships between *N_e_u* and genomic attributes (Table 1) are negative and thus are at least qualitatively consistent with the Lynch & Conery model, suggesting that power may be an issue. Furthermore, given that the *N_e_u* estimates in the current analysis required sequence data, species with small genomes relative to averages within clades are likely overrepresented; thus it would be important to ensure that species with large genomes are included in future analyses. Third, future studies would benefit from more robust estimates of genetic drift, as *N_e_u* estimated from silent-site diversity (as in [7] and the present reanalysis) has several undesirable properties. Because the mutation rate *u* differs among lineages [11], [23], [24], using *N_e_u* as a proxy for *N_e_* could obscure any relationship between *N_e_* and genome size. Further, *N_e_* estimated from silent-site diversity may signal the effects of recent evolutionary events more than the long-term history under which genome size evolved [11]. K_a_/K_s_ ratios (ratios of nonsynonymous to synonymous substitutions per site) are a promising alternative to *N_e_u* for estimating genetic drift [11], [20]. Finally, genome size is a complex trait that is unlikely to be explained by univariate analyses [10]. Phylogenetic comparative methods should be combined with multivariate models that are capable of distinguishing the contributions of highly correlated predictor variables. A recent analysis [19] is a step in the right direction: plant outcrossing rate and *N_e_* were simultaneously examined in a multiple regression analysis of phylogenetically independent contrasts, allowing the partial contribution of each variable to be characterized. To make further progress on the population genetics of genome size and complexity, we clearly need phylogenetic comparative analyses of large datasets capable of distinguishing the contributions of *N_e_* and its multiple correlates, including body size, developmental rate, and metabolic rate.

## Materials and Methods

### Data sources

Data on *N_e_u* and genome sizes for 22 eukaryotic and 7 prokaryotic species were obtained from the Supporting Online Material of [7]. For a subset of these species, data on gene number, intron size, intron number, and the half-life of gene duplicates were also obtained from the same source. Data on total transposon number and fraction of the genome occupied by transposons were obtained directly from M. Lynch; these data combine counts of LTR, non-LTR, and DNA transposons and correspond to the fourth panel of Fig. 4 of [7]. All traits were log_10_ transformed prior to analysis; for total transposon number and transposon fraction, constants of 1.0 and 0.01, respectively, were added prior to log-transformation.

### Phylogeny construction

A composite tree for the species was constructed in Mesquite v. 2.71 [25] based on phylogenetic trees reported in [26]–[28]. As a visual heuristic, genome sizes were traced onto the phylogeny using the Parsimony Ancestral States method [29] with an assumption that all branch lengths equal 1.0.

### Phylogenetic comparative analyses

All dependent variables were regressed on *N_e_u* using REGRESSIONv2.m [30] running in MATLAB v. 7.9.0. Three types of models were examined: ordinary least squares (OLS), phylogenetic generalized least squares (PGLS), and phylogenetic regression under an Ornstein-Uhlenbeck process (RegOU) [30], [31]. OLS is traditional ‘nonphylogenetic’ regression, which in effect assumes a star phylogeny in which all species are equally unrelated, and corresponds to the *N_e_u* vs. genome size analysis reported in [7]. PGLS assumes that residual variation among species is correlated, with the correlation given by a Brownian-motion like process along the specified phylogenetic tree (topology and branch lengths). PGLS is functionally equivalent to Felsenstein's [15] phylogenetically independent contrast method [31]. Finally, the RegOU model estimates (via restricted maximum likelihood) the strength of phylogenetic signal in the residual variation simultaneously with the regression coefficients; the former is given by *d*, the Ornstein-Uhlenbeck transformation parameter. An OU evolutionary model is typically used to model the effects of stabilizing selection around an optimum [30]. When *d* = 0, there is no phylogenetic signal in the residuals from the regression model; when *d* is significantly greater than 0, significant phylogenetic signal exists [30], [32].

Following [33], starter branch lengths corresponding to all branches  = 1.0, Grafen's arbitrary lengths, Pagel's arbitrary lengths, and Nee's arbitrary lengths were compared in PGLS and RegOU regressions of genome size on *N_e_u*. Based on their likelihoods, the models with all branches  = 1.0 achieved the best fit, and thus these branch lengths were used in all subsequent phylogenetic analyses. Model selection for each variable then proceeded in two steps. First, we compared the likelihoods of the PGLS model and the OLS model, with a higher likelihood taken as evidence of a better-fitting model. Second, we used ln likelihood ratio tests (LRTs) to compare the RegOU model with the PGLS and OLS models with 1 d.f. [30]. Given the issue of small sample sizes (see [32]) for most dependent variables and the fact that RegOU models require estimation of an extra parameter, RegOU models were examined only for genome size and gene number.
